# Sex Differences in Psychological Status and Fatigue of Frontline Staff After the COVID-19 Outbreak in China: A Cross-Sectional Study

**DOI:** 10.3389/fpsyg.2021.676307

**Published:** 2021-09-08

**Authors:** Ziwei Teng, Yuhan Su, Jindong Chen, Renrong Wu, Hui Tang, Haishan Wu, Xuming Liu, Heqiao Ling, Hui Yuan, Jing Huang

**Affiliations:** ^1^Department of Psychiatry, The Second Xiangya Hospital, Central South University, Changsha, China; ^2^Hunan Key Laboratory of Psychiatry and Mental Health, Chinese National Clinical Research Center for Mental Disorders (Xiangya), Chinese National Technology Institute on Mental Disorders, Mental Health Institute of the Second Xiangya Hospital, Central South University, Changsha, China; ^3^Department of Intensive Care Unit, Hunan Provincial Hospital of Traditional Chinese Medicine, Zhuzhou, China; ^4^Department of Psychiatry, Doumen Qiaoli Hospital of Traditional Chinese Medicine, Zhuhai, China; ^5^Department of Ultrasound Dltrasound Diagnosis, The Second Xiangya Hospital, Central South University, Changsha, China

**Keywords:** COVID-19, sex difference, psychological status, fatigue, frontline staff

## Abstract

**Background:** The coronavirus disease 2019 (COVID-19) is spreading globally, and it is significant to pay attention to the mental health of frontline staff in this pandemic. This study is aimed to explore the sex difference among the frontline staff in demographics, characteristics of mental state, and the potential relationship between them.

**Method:** A total of 2,614 Chinese frontline staff were recruited. The Self-Rating Anxiety Scale (SAS) and the Patient Health Questionnaire-9 (PHQ-9) were used for assessing the mental status of frontline staff, and the Fatigue Self-Assessment Scale (FSAS) was used for detecting fatigue.

**Result:** The prevalence rate of anxiety for female frontline staff is higher than that of male (*P* = 0.003), and the prevalence rate of depression is similar between them (*P* = 0.091). After comparing the risk factors of unhealthy mental state between different sexes, it is found that family income below 100,000 [depression: odds ratio (OR) 1.37; 95% CI, 1.08–1.73; anxiety: OR 1.99; 95% CI, 1.44–2.75], unsupported of family (depression: OR 10.94; 95% CI, 1.39–85.79; anxiety: OR 11.92; 95% CI, 3.80–37.36), and marriage (depression: OR 1.67; 95% CI, 1.15–2.43) are risk factors for male, and total fatigue (depression: OR 2.96; 95% CI, 1.46–6.02) is risk factor for female.

**Conclusions:** This study found that depression and anxiety are widespread among the frontline staff of COVID-19, and anxiety showed a higher prevalence rate among female frontline staff. From the sex difference in risk factors, the focus of psychological interventions may differ between genders. Men with low family income, unsupported by family or marriage, and women with a high score of total fatigue required particular attention to their psychological status.

## Background

The coronavirus disease 2019 (COVID-19) caused by the severe acute respiratory syndrome coronavirus 2 (SARS-CoV-2) is speeding globally. Up to December 18, 2020, 73,275,943 confirmed cases and 1,650,348 death cases in the world are reported by the WHO [W.H.O. (WHO)., 2020]. In this outbreak, it is significant to pay attention to the mental health of frontline staff. Like other public health emergencies, such as acute respiratory syndrome coronavirus (SARS-CoV), the COVID-19 epidemic may also cause psychological reactions, such as emotional distress, maladaptive behaviors, and defensive responses (Pfefferbaum and North, 2020; Xiang et al., 2020). According to a large web-based survey on 8,177 students, poor housing is associated with an increased risk of depressive symptoms during lockdown (Tang et al., 2014). These reactions are translated by the negative effect that public health emergencies influence the well-being, health, and safety of individuals.

Since travel to and from Wuhan was restricted on January 23, frontline staff, such as doctors, nurses, polices, volunteers, community workers, and journalists, have made a great contribution to effectively controlling the spread of COVID-19. Under the high risk of infection and stress of work, it is easy for frontline staff to experience anxiety, depression, and fatigue. Early research focused on the mental health of frontline medical workers. Depression (73.91%), anxiety (60.14%), and insomnia (43.48%) are generally experienced by the frontline medical workers in Hubei province (Liang et al., 2020). In addition, other frontline staff, such as community workers and volunteers, may also experience abnormal mental states. From the data of a cross-sectional study in October 2020 that focus on the evaluation of psychological status for 2,614 frontline staff in China, the frontline staff was experiencing depression (50%), anxiety (23.4%), and symptom of fatigue (75.7%) (Teng et al., 2020). These prevalent mental health problems highlight the importance of psychological counseling for frontline staff in the fight against COVID-19.

Previous studies found that female frontline staff experienced more serve mental fatigue and anxiety (Batra et al., 2020; Teng et al., 2020; Duplaga and Grysztar, 2021). Although the reason for the sex difference is not clear, there are also some factors related to it. Sociocultural factors may be related to the sex difference in an unhealthy mental state. For example, Kang et al. (2020) found that female participants experienced more severe depression than male participants due to their lower education level and experience of recurrent depressive episodes. Additionally, biological factors may also lead to sex differences. Nevertheless, few studies have explored the sex difference in demographics, characteristics of mental state, and the potential relationship between them, which is the aim of this study and may be helpful for psychological counseling for frontline staff.

## Method

### Participants

This study used a cross-sectional design to explore the sex difference in demographics and characteristics of the mental state of Chinese frontline staff in COVID-19. A total of 2,614 Frontline staff were recruited from March 1, 2020, to March 15, 2020. All the participants were invited to complete this survey by a web link linked to a questionnaire in the Chinese language. It included three parts: informed consent, personal information, and self-reported scales. As the first part, informed consent will be explained to participants, i.e., the aim of this study and the protection of their privacy, so that they can consider whether they would take part in this survey. Personal information includes gender, age, type of work, community, education level, health condition, family income, and marital status. The symptoms of anxiety, depression, and fatigue will be evaluated in self-reported scales.

### Self-Reported Scales

Anxiety, depression, and fatigue are the three main factors for examining the psychological state of participants. The Self-Rating Anxiety Scale (SAS) (Zung, 1965) is used to quantify anxiety, which consists of 20 statements. Each of the statements is scored on a scale of 1–4 to present the frequency of the symptom. The standard score is derived by multiplying the original score, the total score of 20 answers, by 1.25. According to the standard score, anxiety can be divided into three levels: severe anxiety (70 and above), moderate anxiety (60–69), and mild anxiety (50–59).

The presence and severity of depression are assessed by Patient Health Questionnaire-9 (PHQ-9) (Kroenke et al., 2001). It contains nine items according to the Diagnostic and Statistical Manual of Mental Disorders-IV (DSM-IV) diagnostic criteria of depression. Every item is scored on 0–3 points to present the frequency of it. The evaluation criteria were severe depression (15 and above), moderate depression (10–14), mild depression (5–9), and normal range (0–4).

Fatigue Self-Assessment Scale (FSAS) (G.C.o.C.A.o.C. Medicine, 2019) is used to detect the fatigue of participants. It evaluates characteristics, type, and extent of fatigue through 23 items, of which the first 22 items are scored on 0–4 points, and the last is about the changes in fatigue severity over different time periods of the day.

### Data Analysis

The data were analyzed *via* IBM SPSS Statistics for MAC software. Kolmogorov–Smirnov one-sample test was used to measure the normal distribution of continuous variables. Demographics and features of mental state were compared between male and female frontline staff with the chi-square test that was used for categorical variables and Mann–Whitney U tests for continuous variables. After adjusting for confounding variables of each sex, the odds ratios (ORs) were calculated by binary logistic regression and were performed between frontline staff with depression, non-depression, anxiety, and non-anxiety. The SAS scores ≥ 50 were determined as anxiety, and PHQ-9 scores ≥ 5 were determined as depression. The significance level was set at *P* = 0.05, and all tests were two-tailed.

## Results

### The Characteristics of Participants in Demographics and Characteristics of the Mental State of Chinese Frontline Staff in COVID-19

Demographics and information of the mental state of 2,614 frontline staff, such as community workers, healthcare workers, volunteers, market administrations, and others (journalists, police, and commanders), have been collected. There are remarkable differences in several variables between male and female sex. More male participants worked as community workers, volunteers, and others than female participants. In addition, male participants are more likely to have a history of mental or physical disorders (*P* < 0.001), family income below 100,000 RMB (*P* = 0.023), smoking (*P* < 0.001), and focus on epidemic-related situations over 3 h each day (*P* = 0.021). On the contrary, female participants are more likely to come from urban cities, be worried about infection, and report sleep difficulty (Table 1). Moreover, from the outcome of self-reported scales, female participants are more likely to experience anxiety (*P* = 0.003), and the proportion of their score on total (*P* = 0.004), physical fatigue (*P* = 0.003), and mental (*P* < 0.001) fatigue that above the cutoff point is more than that of male participants. It is notable that although 20.7% of male and 25.6% of female participants have symptoms of depression, the difference between them is not statistically significant (*P* = 0.091).

**Table 1:** The characteristics of participants in Chinese frontline staff in COVID-19.

**Variables**	**Male 1,161 (44.4)**	**Female1,453 (55.6)**	**χ2**	**df**	***P***
**Frontline staff**					
Community workers	255 (22.0)	465 (21.1)	182.831	4	<0.001
Healthcare workers	96 (8.3)	302 (32.0)			
Volunteers	351 (30.2)	209 (20.8)			
Market administrations	122 (10.5)	170 (14.4)			
Others^a^	337 (29.0)	307 (11.70)			
**Community**					
Rural	95 (10.1)	119 (6.7)	9.935	1	0.002
Urban	1,066 (89.9)	1,334 (93.3)			
Physical or mental disease	223 (19.2)	195 (13.4)	16.088	1	<0.001
**Education**					
Below university	155 (13.4)	157 (10.8)	5.178	2	0.075
College	963 (82.9)	1,252 (86.2)			
Master's or doctorate	43 (3.7)	44 (3.0)			
Family income (RMB), <100,000	733 (63.1)	854 (58.8)	5.144	1	0.023
**Marital status**					
Single	202 (17.4)	256 (17.6)	1.021	2	0.6
Married	899 (77.4)	1,109 (76.3)			
Others^b^	60 (5.2)	88 (6.1)			
Smoking, n (%)	606 (52.2)	40 (2.8)	847.937	1	<0.001
**How long does it take each day to focus on epidemic related situations**					
<1 h	255 (22.0)	368 (25.3)	9.7720	3	0.021
1–3 h	405 (34.9)	540 (37.2)			
3–6 h	110 (9.5)	131 (9.0)			
> 6 h	391 (33.7)	414 (28.5)			
Worried about being infected	886 (76.3)	1,196 (82.3)	14.327	1	<0.001
Family supports your participation in epidemic prevention	1,150 (99.1)	1,441 (99.2)	0.109	1	0.741
The people you serve are satisfied with your work	1,143 (98.4)	1,440 (99.1)	2.368	1	0.124
Sleep difficulty	567 (48.8)	807 (55.5)	11.629	1	0.001
Depression	559 (48.1)	748 (51.5)	2.865	1	0.091
Anxiety	240 (20.7)	372 (25.6)	8.749	1	0.003
Sleep Unchanged
Total fatigue	848 (73.0)	1,132 (77.9)	8.322	1	0.004
Physical fatigue	754 (64.9)	1,022 (70.3)	8.618	1	0.003
Mental fatigue	832 (71.7)	1,130 (77.8)	12.859	1	<0.001

a *includes commanders, police, and journalists.*

b *includes divorced and widowed*.

### Sex Difference in Anxiety, Depression, and Fatigue Scores

**Figure 1:**
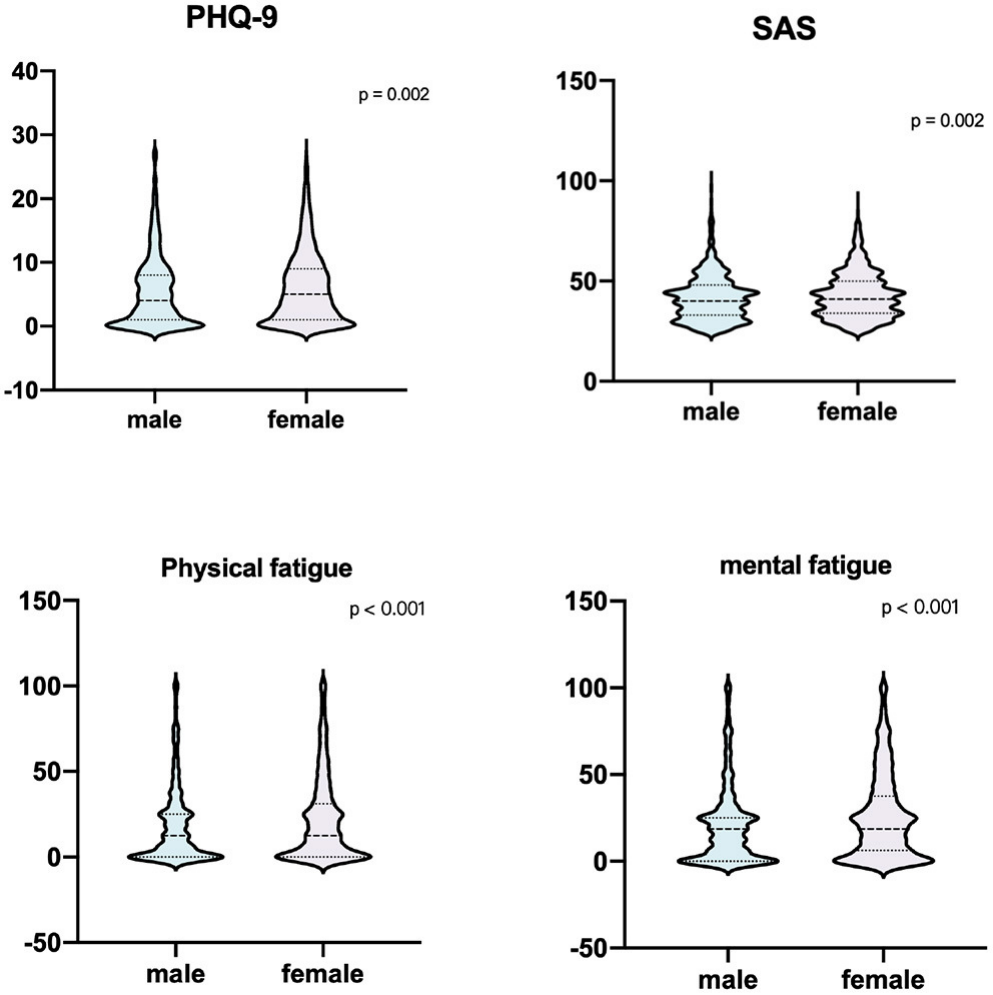
Gender differences in the total scores of PHQ-9, SAS, physical fatigue, and mental fatigue. Female PHQ-9, SAS, physical fatigue, and mental fatigue (blue) presented significantly higher scores in all four scales when compared with males (purple) (*P* < 0.001). Violin plots display the distribution of scale scores. The boxplots within the violins represent the median (the horizontal line in the box), first and third quartiles (box edges). PHQ-9, patient health questionnaire-9; SAS, the self-rating anxiety scale.

Figure 1 shows sex differences in characteristics of mental state associated with depression, anxiety, physical fatigue, and mental fatigue. Women had significantly higher scores in all these scales when compared with men. (Median [the interquartile range, IQR] scores in males vs. females: PHQ-9, 4.0 [1.0–8.0] vs. 4.0 [1.0–9.0]; SAS, 40 [32.5–47.5] vs. 50 [41.25–58.75]; physical fatigue, 12.5 [0.0–25.0] vs. 12.5 [0.0–31.25]; mental fatigue, 18.75 [0.0–25.0] vs. 18.75 [6.25–18.75]; all *P* < 0.01).

### Sex Difference in Risk Factors and Psychological Impact

After controlling for confounding factors in Table 2, the multivariable logistic regression analysis shows that in male participants, community workers are associated with more severe depression compared with other occupation frontline staff (OR 1.75; 95% CI, 1.25–2.43; *P* < 0.05). In contrary, healthcare workers are associated with less serve symptom of depression (OR 0.54; 95% CI, 0.34–0.87; *P* < 0.05) and anxiety (OR 0.42; 95% CI, 0.21–0.85; *P* < 0.05). Similarly, market administrations are also associated with fewer serve symptoms of depression (OR 0.57; 95% CI, 0.37–0.88; *P* < 0.05). The relationship between the type of occupation and psychological status of female participants is similar to that of male participants. It should be added that female community workers are also associated with more severe anxiety (OR 1.83; 95% CI, 1.33–2.52; *P* < 0.05) and less severe symptoms of anxiety (OR 0.36; 95% CI, 0.22–0.63; *P* < 0.05) than other women.

**Table 2:** Demographics and variables of mental state independently associated with depression and anxiety in each sex.

	**Male**		**Female**	
**Variables**	**Depression**	**Anxiety**	**Depression**	**Anxiety**
**Frontline staff**				
Community workers	**1.75 (1.25, 2.43)**	1.31 (0.90,1.92)	**1.89 (1.41, 2.55)**	**1.83 (1.33, 2.52)**
Healthcare workers	**0.54 (0.34, 0.87)**	**0.42 (0.21, 0.85)**	**0.52 (0.37, 0.72)**	**0.53 (0.35, 0.79)**
Volunteers	0.99 (0.73, 1.33)	0.85 (0.58, 1.23)	0.92 (0.65, 1.31)	0.92 (0.61, 1.39)
Market administrations	**0.57 (0.37, 0.88)**	0.79 (0.47, 1.35)	**0.41 (0.27, 0.60)**	**0.36 (0.22, 0.63)**
Others^a^	Reference	Reference	Reference	Reference
**Age (years)**				
18–24	**2.61 (1.22, 5.55)**	1.42 (0.59, 3.41)	**8.27 (1.65,41.34)**	5.53 (0.67,45.51)
25–34	**2.44 (1.44, 4.13)**	1.42 (0.76, 2.67)	**6.13 (1.32,29.99)**	3.67 (0.46, 29.24)
35–54	1.39 (0.81, 2.23)	0.89 (0.48, 1.65)	2.93 (0.62, 13.93)	3.67 (0.46, 29.24)
55–64	Reference	Reference	Reference	Reference
**Residence**				
Rural	1.15 (0.78,1.68)	1.37 (0.88,2.13)	1.43 (0.94, 2.18)	1.54 (0.99, 2.39)
Urban	Reference	Reference	Reference	Reference
**Education**				
Below university	0.85 (0.43, 1.67)	0.88 (0.40,1.92)	0.25 (1.25, 0.51)	**0.37 (0.17, 0.81)**
College	0.88 (0.48, 1.63)	0.73 (0.36, 1.47)	0.66 (0.36, 1.23)	0.88 (0.45, 1.71)
Master's or doctorate	Reference	Reference	Reference	Reference
Physical or mental disease	**3.26 (2.37, 4.48)**	**3.20 (2.32, 4.40)**	**3.67 (2.59, 5.22)**	**3.91 (2.86, 5.34)**
Family income (RMB) <10,0000	**1.37 (1.08, 1.73)**	**1.99 (1.44, 2.75)**	1.22 (0.98, 1.50)	**1.78 (1.38, 2.29)**
**Marriage**				
Single	Reference	Reference	Reference	Reference
Married	**1.67 (1.15, 2.43)**	0.95 (0.49. 1.79)	**0.39 (0.29, 0.52)**	**0.55 (0.41, 0.73)**
Others^b^	0.75 (0.54, 1.04)	0.58 (0.32, 1.04)	**0.50 (0.31, 0.82)**	0.81 (0.48.1.37)
**Time focus on epidemic related situations**				
<1 h	Reference	Reference	Reference	Reference
1–3 h	0.88 (0.64, 1.21)	1.08 (0.70, 1.66)	0.89 (0.68, 1.16)	0.97 (0.69, 1.36)
3–6 h	1.41 (0.89, 2.21)	**2.27 (1.33, 3.87)**	1.13 (0.76, 1.68)	1.53 (0.96, 2.42)
> 6 h	**1.48 (1.08, 2.04)**	**1.981 (1.32, 2.98)**	**2.05 (1.53, 2.72)**	**2.49 (1.80, 3.45)**
Worried about being infected	**1.78 (1.23, 2.55)**	**3.97 (2.48,6.35)**	**2.57 (1.93, 3.41)**	0.95 (0.62, 1.45)
Family don't support your participation in epidemic prevention	**10.94 (1.39, 85.79)**	**11.92 (3.80, 37.36)**	6.82 (0.77, 59.89)	**7.67 (1.48, 39.82)**
The people you serve are not satisfied with your work	**18.85 (2.50, 142.1)**	**7.41 (1.82, 30.25)**	**11.64 (1.31, 103.63)**	**3.90 (1.04, 14.52)**
Sleep difficulty	**9.71 (7.42, 12.70)**	**12.64 (8.29,19.27)**	**8.13 (6.39, 10.35)**	**6.81 (4.66, 9.94)**
Physical fatigue	**6.52 (4.22, 10.08)**	**36.88 (8.97, 151.74)**	**4.92 (3.30, 7.33)**	**12.68 (5.22, 30,85)**
Mental fatigue	**3.83 (2.02, 6.68)**	**14.10 (3.38, 58.77)**	**6.43 (3.57,11.59)**	**29.24 (3.78, 226.06)**

a *includes commanders, police, and journalists.*

b *includes divorced and widowed. The bold values means p < 0.05*.

Education level may also influence the psychological status of women. Higher education level is associated with a higher score on anxiety (e.g., severe anxiety in master or doctorate vs. below university, OR 0.37; 95% CI, 0.17–0.81; *P* < 0.05), which is only confirmed among female participants. It is worth noting that marriage has an opposite effect on psychological status. Compared with single male participants, that married is associated with more severe depression (OR 1.67; 95% CI, 1.15–2.43; *P* < 0.05). In contrary, married women are related to lower score on depression (OR 0.39; 95% CI, 0.29–0.53; *P* < 0.05) and anxiety (OR 0.55; 95% CI, 0.41–0.73; *P* < 0.05) than single woman, and other marital status women are scored lower on depression (OR 0.50; 95% CI, 0.31–0.82; *P* < 0.05).

Furthermore, there are several remarkable variables associated with higher score on depression and anxiety in male participants, such as history of physical or mental disease (OR 3.26; 95% CI, 2.37–4.48; *P* < 0.05; OR 3.20; 95% CI, 2.32–4.40; *P* < 0.05), family income (RMB) below 100,000 (OR 1.37; 95% CI, 1.08–1.73; *P* < 0.05; OR 1.99; 95% CI, 1.44–2.75; *P* < 0.05), worries about infection (OR 1.78; 95% CI, 1.23–2.55; *P* < 0.05; OR 3.97; 95% CI, 2.48–6.35; *P* < 0.05), unsupported participation in epidemic prevention of family (OR 10.94; 95% CI, 1.39–85.79; *P* < 0.05; OR 11.92; 95% CI, 3.80–37.36; *P* < 0.05), dissatisfaction of service people (OR 18.85; 95% CI, 2.50–142.1; *P* < 0.05; OR 7.41; 95% CI, 1.82–30.25; *P* < 0.05), sleep difficulty (OR 9.71; 95% CI, 7.42–12.70; *P* < 0.05; OR 12.64; 95% CI, 8.29–19.27; *P* < 0.05), physical fatigue (OR 6.52; 95% CI, 4.22–10.08; *P* < 0.05; OR 36.88; 95% CI, 8.97–151.74; *P* < 0.05), and mental fatigue (OR 3.83; 95% CI, 2.02–6.68; *P* < 0.05; OR 14.10; 95% CI, 3.38–58.77; *P* < 0.05).

In female participants, history of physical or mental disease (OR 3.67; 95% CI, 2.59–5.22; *P* < 0.05; OR 3.91; 95% CI, 2.86–5.34; *P* < 0.05), dissatisfaction of service people (OR 11.64; 95% CI, 1.31–103.63; *P* < 0.05; OR 3.90; 95% CI, 1.04–14.52; *P* < 0.05), sleep difficulty (OR 8.13; 95% CI, 6.36–10.35; *P* < 0.05; OR 6.81; 95% CI, 4.66–9.94; *P* < 0.05), physical fatigue (OR 4.92; 95% CI, 3.30–7.33; *P* < 0.05; OR 12.68; 95% CI, 5.22–30.85; *P* < 0.05), and mental fatigue (OR 6.43; 95% CI, 3.57–11.59; *P* < 0.05; OR 29.24; 95% CI, 3.78–226.06; *P* < 0.05) are associated with more severe depression and anxiety. In addition, worries about infection (OR 2.57; 95% CI, 1.93–3.41; *P* < 0.05) and total fatigue (OR 2.96; 95% CI, 1.46–6.02; *P* < 0.05) are also related to higher score on depression, and family income (RMB) below 1,00,000 (OR 1.78; 95% CI, 1.38–2.29; *P* < 0.05) and unsupported participation in epidemic prevention of family (OR 7.67; 95% CI, 1.48–39.82; *P* < 0.05) are related to higher score on anxiety.

## Discussion

There are three main findings in this study: (1) the prevalence rate of anxiety for female frontline staff is higher than that of male staff, and the prevalence rate of depression is similar between them; (2) male participants with depression, anxiety, or fatigue scores are lower than that of female; (3) male participants with the unhealthy mental state are associated with low family income, unsupported of family, and marriage, and female participants with the unhealthy mental state are associated with total fatigue and education level.

Moreover, this study also found that family factors might significantly influence men, which may be related to the role of men in a family. In Chinese traditional culture, men always are the spiritual and economic pillars of a family, which means that men may experience more pressure from economic aspects than women. Therefore, it may be the reason why low family income is one of the risk factors of abnormal mental state for male frontline staff. This finding also serves as a reminder that government should value the distribution of benefits to frontline staff with low family income, which can also benefit their mental health. Furthermore, the support from family is also essential for the frontline staff, especially for men, which points out that the government should also make a good deal of psychological work for the families of frontline staff. Family support may alleviate anxiety and depression that occur in frontline staff. Parent support can remarkably reduce the association between depression and acculturative stress than friends (Crockett et al., 2007; Raffaelli et al., 2013). This benefit of the support may also work when frontline staffs are under stress from their work.

In addition, it is interesting that marital status exerts two diametrically opposite effects on the mental health of frontline staff of both sexes. Compared with single male frontline staff, that married is associated with more severe depression, which is opposite to female frontline staff. The state of being married is associated with a severe and abnormal mental state for men, and the state of being single is associated with a severe abnormal mental state for females. This difference may be related to the different influences brought by family on men and women. Women with negative emotions may be more likely to be healed by the love of their husbands or children from their marriage. Differently, some previous studies pointed out that the family seems like a promoting factor of depression for women. Because men and women play different roles in the family that women are more likely to be in charge of childcare and household duties than women, the work–family conflict for females may increase (McHenry et al., 2014; Guille et al., 2017). This conflict produced by the unequal division of domestic labor may benefit the prevalence of depression. However, the situation may be changed recently. The household and childcare duties are shared by both men and women in the family, which means that the work–family conflict of women may reduce compared with the past.

Sex differences in psychological status and fatigue are common.

This study also found that for female frontline staff, high education level is one of the risk factors of unhealthy mental state, which is inconsistent with some previous studies. These previous studies showed that a lower education level is associated with postpartum depression (Matsumura et al., 2019), and a cross-sectional study in Europe found that the higher education level decreases the prevalence of depression (Freeman et al., 2016). The difference between this study and previous studies suggests that the psychological status of frontline staff with high education levels may need more attention, especially the female frontline staff.

The higher prevalence rate of anxiety may be related to sex hormonal fluctuation. The low level of progesterone and oestradiol associated with menstruation, menopause, or postpartum may decrease the synthesis of allopregnanolone and serotonergic, the endogenous anxiolytics (Li and Graham, 2017). It means that women have a decreased level of protective hormone for anxiety during specific physiological periods making them more likely to experience symptoms of anxiety. In addition, women may be more likely to be influenced by the downregulation of allopregnanolone or abnormal serotonin induced by stress (Li and Graham, 2017). It suggests that women may be more likely to experience anxiety if both men and women experience the same hormonal changes simultaneously. It is worth noting that the findings of the effect of sex hormones on anxiety are based on the general living environment, which means that the effect of sex hormonal fluctuation may not wholly explain the sex difference in the anxiety of this study. A particular working environment may be associated with stress that may influence the changes in sex hormone levels. Furthermore, the similar prevalence of depression in male and female participants found in this study is also mentioned in the previous study. A cross-sectional study exploring the mental health of medical university students also found that although female students are presented with the increasing prevalence of stress compared to male students, and there is no significant difference in depression between them (Yusoff et al., 2013). However, some studies have found the sex difference in depression that from adolescents to adults, and men are about half as likely to be diagnosed with depression as women (Girgus and Yang, 2015). Under stress, it is found that more depression is reported in girls than boys (Hankin et al., 2007; Girgus and Yang, 2015). The higher reported stress-induced depression in girls shows that female frontline staff may also have more severe stress-induced depression than male staff. Nevertheless, it should be noticed that the sex difference in depression among teenager participants may not be appropriate to extrapolate the sex difference among frontline staff due to the impact of adolescence.

In this study, we suggested that anxiety and depression in men can be alleviated by increasing their income. Men unsupported by family or marriage and women with high education levels should be given special psychological attention, such as conducting regular psychological assessments or visiting local psychiatric departments to ask for help.

There are several limitations of this study. First, because of the cross-sectional study, causality and trend in psychological change cannot be represented in this study, which suggests that future research directions could inform follow-up investigations of frontline staff after therapeutic interventions. Second, one of the major limitations of a cross-sectional study and online study is selection bias, and the participants included can be poorly representative and cannot represent the target population. In our study, most participants of this study were recruited from Hunan province, which makes the population cannot represent the national data. Although we conduct an online survey, we cooperated with the local government to collect questionnaires. The first-line staff participated in this study when they were attending meetings organized by the government or in their WeChat Workgroup through scan QR code. It means that the majority of the local first-line staff were included.

## Conclusions

This study found that depression and anxiety were widespread among the frontline staff of COVID-19, and anxiety had a higher prevalence rate. It is also found that there are some differences in risk factors of abnormal mental state between genders. From that, it is possible that the focus of psychological interventions may differ between genders. Men with low family income, unsupported by family or marriage, and women with high education levels require particular attention to their psychological status.

## Data Availability Statement

The original contributions presented in the study are included in the article/supplementary material, further inquiries can be directed to the corresponding author/s.

## Ethics Statement

The studies involving human participants were reviewed and approved by the Ethics Committee of the Second Xiangya Hospital of Central South University. The patients/participants provided their written informed consent to participate in this study.

## Author Contributions

All authors contributed to and approved the final manuscript.

## Funding

This study was supported by grants from the National Natural Science Foundation of China (grant no. 81971258 and 81901401).

## Conflict of Interest

The authors declare that the research was conducted in the absence of any commercial or financial relationships that could be construed as a potential conflict of interest.

## Publisher's Note

All claims expressed in this article are solely those of the authors and do not necessarily represent those of their affiliated organizations, or those of the publisher, the editors and the reviewers. Any product that may be evaluated in this article, or claim that may be made by its manufacturer, is not guaranteed or endorsed by the publisher.
